# A cascade of causes that led to the COVID-19 tragedy in Italy and in other European Union countries

**DOI:** 10.7189/jogh-10-010335

**Published:** 2020-06

**Authors:** Igor Rudan

**Affiliations:** Centre for Global Health, Usher Institute, University of Edinburgh, Edinburgh, Scotland, UK

The perception of the COVID-19 pandemic in my homeland Croatia has been based on two main sources of information over the past three weeks. On the one hand, our Civil Protection Headquarters, as well as all of the experts and scientists to whom media space has been provided, called for caution, but without any panic. They emphasized that this was not a cataclysm, but an epidemic involving a serious respiratory infectious disease. The cause of this disease is the new coronavirus, for which we do not have a vaccine. Therefore, it can be expected that the disease will be very dangerous for the elderly and to those who are already ill. So, it was an unknown danger worthy of caution, but our epidemiologists remained calm. They knew that they would be able to estimate the epidemic’s development using data and then control the situation with anti-epidemic measures, and through several lines of defence [1].

On the other hand, the people of Croatia also followed the events in Italy. From there, day after day, apocalyptic news came, with incredibly large numbers of infected and dead. Daily reports from Italy seemed completely incompatible with what the experts and scientists in Croatia were saying. Some have concluded that a scenario similar to that in Italy, if not worse, is inevitable for Croatia. The population was in a very confusing situation.

In this text, I will try to penetrate the very core of the “infodemia” that has been present in the media across many European countries, as well as on social networks, over the past three weeks. I will explain how that disturbing situation arose and offer a scientific explanation for it. This seems important at this point, because the Italian tragedy with the COVID-19 epidemic has, unfortunately, hindered the credible and scientifically-based communication of the epidemiological profession to the population of Croatia.

**Figure:**
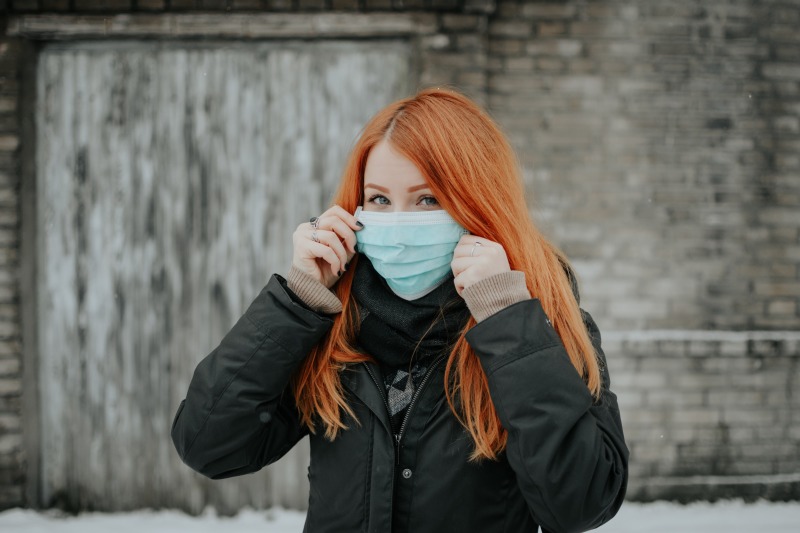
**Photo:** Photo by Pille-Riin Priske on Unsplash.

In my article “20 Key Questions and Answers on Coronavirus” posted on the 9^th^ of March, 2020 on Index.hr [1], in answer to question number 18, “With the effectiveness of quarantine in China, can we draw some lessons from this pandemic?”, I stated:

“If the virus continues to spread throughout 2020, it will demonstrate in a very cruel way how well the public health systems of individual countries are functioning… These will be very important lessons in preparation for a future pandemic, which could be even more dangerous.”

We are now slowly entering a phase where many countries have been exposed to the pandemic for sufficiently long to be able to make some first estimates of their results. From these days up until the end of the pandemic, we will see that COVID-19 will divide the world into countries that have relied on epidemiology and followed the maths and the logic of the epidemic, as well as those in which this isn’t the case, and many sadly, probably quite unnecessarily — will suffer.

An epidemic is a serious threat to entire nations, during which citizens’ interest in other topics may vanish quite rapidly. We could see that happening quite clearly in the past several weeks. The task of epidemiologists is to constantly have tables in front of them with a large number of epidemic parameters, reliable field figures and formulas to monitor the epidemic’s development, and to know the ‘’laws” of epidemics, in order to organize the implementation of anti-epidemic measures in a timely manner and thus protect the population.

Now, let’s look at the countries that we can already point out as being successful in their response to this new challenge. First and foremost, there is China. It has completely suppressed the huge epidemic in Wuhan, which spread to all thirty of its provinces. In doing so, it relied on the advice of its epidemiologic legend, 83-year-old Zhong Nanshan. Twenty years ago, Nanshan gained authority by suppressing SARS. Although surprised by the epidemic, they managed to suppress COVID-19 throughout China through expert and determined measures. They did so over just seven weeks, with the death toll eventually coming to a halt at less than 4000 [2]. By comparison, it would be as if the number of deaths in Croatia as a result of this epidemic was kept at around 14 in total.

Furthermore, if at some point you find yourself caught up in the uncertainty surrounding the danger of COVID-19, you should be able to learn more if you look at the state of things in Singapore. Despite intensive exchanges of people and goods with China since the outbreak of the epidemic, Singapore has a total of 732 infected people as I am writing this article, with two dead and 17 more in intensive care [2]. For a long time, this city-state has been nurturing the ambition to lead the world in all measurable parameters. From this, it must be concluded that the developments in Singapore are a likely reflection of the real danger of COVID-19 in a country that is based on knowledge, technology, good organization, and general responsibility. Also, Singapore had a relatively recent SARS experience and learned from it, so they built capacity and ensured speed in their response to future challenges. The situation in Singapore, therefore, is an indicator of the effects of the virus on the population, to the extent that it is truly unavoidable.

Any deviation towards something worse than the Singaporean results will be less and less of a consequence of the danger of the virus itself, and increasingly attributable to human omissions. In doing so, human errors that can lead to the unnecessary spread of the infection are: (1) omission to properly understand the epidemic parameters; (2) reluctance to make decisions based on the changes in those parameters; and (3) the irresponsible behavior of the population in complying with instructions from the authorities.

To confirm the statements about Singapore, let’s look at the current situation in other countries that rely on knowledge and expertise and have good organization. They were also the most common destinations for the spread of the epidemic from China in the first wave: Hong Kong, Japan, South Korea, the United Arab Emirates, and Qatar. There were only 519 infected people in Hong Kong at the time of writing this article, with 4 deaths and 5 more people in a serious condition; in Japan 1499 were infected, 49 were dead and 56 were in a serious condition; in South Korea, which had a severe epidemic behind its first line of defence, 9478 were infected, 144 died and 59 were seriously ill; in the United Arab Emirates, 405 were infected, 2 died and 2 were seriously ill; and in Qatar, 562 were infected, 6 were seriously ill, and still no one had died from COVID-19 [2].

Fortunately, Croatia is now up there with all of these countries, with 657 infected, 5 dead and 14 more seriously ill [2]. As you can see pretty clearly from all of these figures, in countries that rely on the knowledge and the profession and properly applied anti-epidemic measures, COVID-19 is a disease that should not be life-threatening for more than a tiny percentage of all infected people. This conclusion can still be reached if it is properly understood that the information on the number of cases and deaths at the website “https://www.worldometers.info/coronavirus” [2] is based on positive tests, and not on everyone who is actually infected. We need to do further research to understand the denominator for the case-fatality rate properly.

What, then, is happening in Italy, as well as in Spain, but to a good extent also in France, Switzerland, Belgium, Austria, Denmark, and Portugal? I did not include Germany, Sweden, the Netherlands, the United Kingdom, and the United States in this group of countries for now. This is because at least for some time during the pandemic, they have clung to the idea of intentionally letting the virus spread and infect at least part of the population.

Given all of the previous examples of a successful epidemiological response, and what is now practically the coexistence of people with the new coronavirus in Asia’s most developed countries, how is it possible for Italy to have nearly 85 000 infected people and over 9000 deaths at the time of this article; or, that Spain has 72 000 infected people and more than 5600 deaths, and France has almost 33 000 infected people and 2000 deaths? Or that even Switzerland, which everyone would expect to see among the most successful countries in any of these world rankings, could already have 13 250 infected people and 240 deaths, with 203 more critically ill people? [2] The causes of all of this are, however, becoming increasingly clear to science.

First of all, there was probably a premature relaxation around the real danger of COVID-19 in Europe. The epidemic development by the end of February was already quite similar to the one seen previously with SARS and MERS. In both of those epidemics, the primary focal point was suppressed. Then, the virus was stopped using the first line of defence – identifying all those infected, tracing their contacts and isolating them all – in more than 25 countries. By the end of February 2020, it was already clear in the case of COVID-19 that it would be successfully suppressed in its primary focal point – Wuhan. It was also already stopped using the first line of defence in another thirty Chinese provinces and surrounding countries in Asia. Then, on the 28^th^ of February, the first estimates of death rates were published, suggesting that it was a disease with a death rate significantly lower than that of SARS and MERS [3,4]. At that time, it was reasonable to expect that the epidemic could soon be stopped. As a result, the World Health Organisation delayed the declaration of a pandemic until the 11^th^ of March, and the world stock markets increased by about 10 percent from the 27^th^ of February to the 3^rd^ of March [5,6]. But for any unknown virus, premature relaxation is very dangerous, as will be shown later with COVID-19.

Second, it is possible that Chinese tourists from Wuhan were visiting Northern Italy in January and February 2020. Fever does not accompany all COVID-19 cases and asymptomatic transmission is possible, so infected tourists could have been allowed to enter Italy even where proper checks at the airports were in place [4]. Italy cancelled flights linking it directly with China on January 31 following the news of the epidemic spread [7]. Some investigative journalists hypothesized, although this has not been confirmed, that it may be possible that the phenomenon of the mass immigration of Chinese workers to northern Italy may have contributed to the early introduction and spread of the virus [8]. Tens of thousands of Chinese migrants work in the Italian textile industry, producing fashion items, leather bags and shoes with the brand “Made in Italy”. They worked in conditions where they were cramped closely together, which would facilitate the spread [8-10]. Reuters and the reporter D. T. Max wrote about this phenomenon back in 2014 and 2018, respectively [9,10]. Partly as a result of this development, direct flights between Wuhan and Italy were introduced. Previous reports claimed that there may be many illegal migrants among those workers [9,10]. Whether through Chinese tourists, textile workers, or through some other route, the novel coronavirus has triggered an epidemic behind the back of the Italian “first line of defence” which remained unrecognized in the first few weeks.

Third, infected people from northern Italy spent their weekends at European ski resorts. Although we do not know if the arrival of the warmer weather will stop the transmission of coronavirus, what we can assume is that the cold helped it to spread. That is why European ski resorts became real nurseries of coronavirus in late February and in early March, as many media reported [11,12]. In this way, more infected people emerged behind the front lines of defence in France, Switzerland, Belgium, Austria, Denmark and Spain. Their first lines of epidemiological defence focused on air transport from Asia, not on their own skiing ‘’returnees”, where indeed no one would expect a large number of Chinese people from Wuhan to be.

Fourth, although Spain may not have had as many skiers as other European countries in this cluster, the virus may have been introduced to them through a “biological bomb”. On the 19^th^ of February, a Champions League football match was held between Italy’s Atalanta and Spain’s Valencia. Atalanta is a team from a small city of Bergamo, Italy, which has 120 000 inhabitants. This was possibly the biggest game in Atalanta’s history, as it progressed through group stages to the last 16 in the European Champions League. The local stadium was not large enough for everyone who wanted to attend the game, so it was moved to a large San Siro stadium in Milan. The official attendance was 45 792, meaning that a third of Bergamo’s population, with around 30 busses, travelled from Bergamo to Milan and then wandered the streets of Milan before the game [13,14]. Unfortunately for Spain, nearly 2500 Valencia fans also travelled to the match. As Atalanta scored four goals, a third of Bergamo’s population was hugging and kissing in the cold weather four times and spent the day closely together. This is likely why it became the worst-hit region of Italy by some distance. Moreover, at least a third of Valencia football squad also got infected with a virus and later played Alaves in the Spanish league, where further players of that team got infected. This football game has certainly contributed to the virus making its way to Spain [14].

Fifth, it is very important for the early development of the epidemiological situation in each country to look at which subset of the population the virus has spread among. Northern Italy has a very large number of very old people. In the early stages of the epidemic, the virus began to spread in hospitals and retirement homes [15,16]. They did not have nearly enough capacities to assist in severe cases. Among already sick, elderly and immunocompromised people, the virus spread more easily and faster and had a significantly higher death rate. In some other countries, such as Germany, most of the patients in the early stages were between the ages of 20 and 60 and were returning from skiing trips or were business people [17]. Therefore, such countries have a significantly lower death rate among those first infected.

Sixth, this is likely the most important factor required to explain the current situation in Italy and other European Union (EU) countries. There must have been either the omission to monitor the mathematical parameters of the epidemic, or perhaps the lack of clear communication of the dangers, or the indecisiveness to adopt isolation measures for the population. It is difficult to know, at this time, which of these three causes is the most important – a combination of them all is entirely possible. However, the nature of the omission largely explains the terrible figures on infections and deaths that are being reported from Italy and other EU countries on a daily basis.

To understand the tragedy in Italy and Europe, we must first return to Wuhan. When the epidemic broke out, the Chinese first had to isolate the virus. Then, they needed to read its genetic code and develop a diagnostic test. It all took time, as the epidemic spread rapidly throughout the city. When they began testing for coronavirus, between the 18^th^ and the 20^th^ of January, they had double-digit numbers of infected people [3]. Those numbers apparently stagnated, so the epidemiologists did not know what that might mean. But on the 21^st^ of January, the number of newly infected people jumped to more than 100. On the 22^nd^ of January, it jumped to more than 200 [3]. This was a clear signal to Chinese epidemiologists that an exponential increase in the number of infected people was occurring. At that time, they had nothing further to wait for, or to think about. If the virus breaks through the first line of defence – and the Chinese didn’t even have any, since the epidemic started there unannounced – then a quarantine measure needs to be triggered. This prevents the virus from spreading further and generating a large number of infected people through the exponential growth of the epidemic.

After such a sudden declaration of quarantine in Wuhan, the huge epidemic wave had actually just begun to show. Everyone who was already infected began to develop the disease in the next several days. The maximum daily number of new infected cases was reached on the 5^th^ of February. On that day alone, as many as 3750 new patients were registered in Wuhan [3]. This means that the “jump” from about 125 to about 250 newly registered infected persons signals to epidemiologists that they should expect an epidemic surge in 14 days, with as many as 3750 newly infected people at the peak.

Let us now explain the “time delay” between people getting infected with a virus and the health system’s ability to detect those infected based on their symptoms. The new coronavirus kills primarily because it spreads incredibly quickly among humans. As a result, it creates a gigantic number of infected and sick in a very short time period (**Table 1**). Among those who are sick, about 5% will require intensive hospital care. If all of them could receive optimal care, we would be able to save nearly everyone. But if they all get very sick at the same time, then we cannot offer adequate care to everyone. As a result, most critical cases will die. This is the main reason why this virus kills so many people in those countries which allowed it to spread freely for too long. This is shown in a simple way in this table, based on day-to-day growth in a number of cases by 26%, which was a very realistic scenario for most EU countries:

**Table 1:** A dynamics of the epidemic of COVID-19 in any given country, based on a realistic scenario of about 26% of day-to-day growth of the number of cases

Time	Number infected	Number sick	Approximate number requiring intensive care
Day 0	1	0	0
Day 7	10	1	0
Day 14	100	10	0
Day 21	1000	100	5
Day 28	10 000	1000	50
Day 35	100 000	10 000	500
Day 42	1 000 000	100 000	5000
Day 49	10 000 000	1 000 000	50 000

With this in mind, let us now look at the Italian and other EU countries’ reaction to their own epidemic, in view of what Croatia did. In the early stages of infection spreading in a country, one or two infected persons are usually detected daily.

Personally, I advised the Croatian authorities and public through social networks to start seriously thinking about social exclusion measures when they noticed a first notable shift from the first 10 confirmed infections towards the first 20 infected people. On the 12^th^ of March, I posted a Facebook status entitled “Contrast is the mother of clarity”, which was viewed and shared by many thousands of my fellow Croatians who have been following my popular science series on the pandemics — “The Quarantine of Wuhan”. This status has also been shared by many online and printed newspapers and media in Croatia, including radio stations [18]. In that status, I suggested that Croatia should consider a large quarantine because we had already jumped from 14 to 19 infected people the day before. However, I also warned them to weigh this decision carefully against economic implications and their expected long-term effects on health. The very next day, on the 13^th^ of March, a decision was made in Croatia to close the schools [18,19].

That meant that, up to that point, Croatia completed two of the most important tasks in this pandemic. The first task was to hold its first line of defence. This was being achieved through the identification of infected cases imported from other countries and their isolation, and that of all their contacts. Croatia completed this first task better than the other EU countries, based on an average percentage increase of cases between the 3^rd^ of March and the 17^th^ of March. Then, from March 13^th^, Croatia also began to introduce social exclusion measures at the right time, thus successfully carrying out the second key task in controlling its own epidemic. Many credits for this should be attributed to its epidemiologists who work at the Croatian Institute for Public Health.

But, what went wrong with these two measures in Italy and other EU countries? On the 21^st^ of February, the number of confirmed infected cases in Italy jumped from 3 to 20. As Italy is a more populous country than Croatia, it might have still been too hasty to send all of Lombardy into quarantine based on this. But on the 22^nd^ of February, the jump was from 20 to 62 cases, and this should lead to some serious thought about social isolation measures. A couple of days later, on the 24^th^ of February, they reached a situation very similar to that in Wuhan before a strict quarantine: the number of confirmed infections jumped from 155 to 229. This was particularly worrying in Italy because they did not seem to proactively test at that time, either.

The “jump” from 155 to 229, in combination with the Wuhan experience, should have suggested that they would have at least 50 000 infected people under the predicted curve of the epidemic wave and they were just seeing its early beginning of it. Such a large number would imply that about 2500 affected would require intensive care units. At the time, Lombardy had only about 500 such units in government/state facilities and another 160 in private health care facilities [20]. As early as the 24^th^ of February it was clear that there would be many deaths in Lombardy weeks later. With epidemics, everything goes awry because the infected get sick a week later, and some of the patients then die ten to twenty days later. This “time delay” is a critically important factor that needs to be taken into account.

However, even then, the Italians did not declare a quarantine. They did not do so on the 29^th^ of February, either, when the total number of infected people rose from 888 to 1128. Those figures implied that in mere days they would be having about 15 000 newly infected people each day. They did not declare quarantine on the 4^th^ of March, either, when the number of infected people exceeded 3000, and when the world stock exchanges started to fall again. It had then become clear to most epidemiologists who have been advising global investors that an unexpected tragedy was about to unravel in Italy and this was now inevitable. At that point, Italy already had at least 30 000 infected people spreading the infection. The quarantine was declared for Lombardy on the 8^th^ of March [7]. The day before, the number of cases had already risen, and exponentially so, to as many as 5883.

To appreciate the problem with epidemic spread in the population behind the first line of defence, this is similar to borrowing €1000 from someone on the 29^th^ of February with an interest rate of 26% each day, meaning an interest rate of 26% on top of that the next day, and so on. Furthermore, there did not seem to be enough clear and decisive communication with the public. The news of the quarantine for Lombardy was, in fact, leaked to the media before it was officially announced. This led to a quick ‘’escape” of many students to the south of the country, to their homes, carrying the contagion with them. As a result, on the 10^th^ of March all of Italy had to be quarantined [7].

In an already difficult situation, where every new day of delay meant another thousand or more people dying, as we can all notice these days, there were numerous media reports warning that the population may not have taken those measures as seriously as the Chinese when they introduced orders to their population in Wuhan [21,22]. Any indiscipline under such grave circumstances could have allowed the virus to take yet another step quite easily. With each new step, another 26% of interest was added to everything before that, and then 26% on everything on everything before that again. That is the power of exponential growth, characteristic of the free spread of the virus in the population.

Many Italians and then Spaniards, as well as residents of several other wealthy countries in Europe, had their lives cut short by their lack of recognition of the dangers of exponential function during the spread of the epidemic. Delaying quarantine for a week made the epidemic ten times worse than it should have been. Delaying it for two weeks made it a hundred times worse. And after two weeks of it being finally proclaimed, all those who may have not taken the orders seriously enough would have made the epidemic several hundred times worse. This means that, in Italy, and possibly in Spain, too, we are now observing the COVID-19 epidemic that is more than a hundred times worse than it should have been in a country that was much better prepared for the response, such as Singapore, Taiwan, Hong Kong or the United Arab Emirates.

To appreciate what is happening in Italy, it is enough to think of this sentence alone: at least 100 times fewer people would die each day if quarantine had been declared 2 weeks earlier and had the population stuck to the recommendations. During those fourteen days between 23^rd^ of February and 7^th^ of March, they allowed the virus to spread freely and infect a huge number of people – maybe even up to a million, or perhaps more, it is very difficult to know at this point. This would mean tens of thousands of people in need of intensive care, with about ten times fewer units available nationwide. About half of those who fall seriously ill will not survive without the necessary support. At this point, whenever we hear that 1000 people died in Italy in one day, we should know that the casualties would only add up to 10 had the quarantine been declared just a couple of weeks earlier. I appreciate that it seems implausible that the delay of a political decision like the introduction of quarantine by just two weeks may mean the difference between 100 deaths and 10 000 deaths in the 21^st^ century. However, I am afraid that this is, unfortunately, the reality of the exponential growth of the number of infected during an epidemic.

What does this mean for the public in countries like Croatia, who were confused and in awe of the events in Italy? They should know that they did not observe what the COVID-19 epidemic should actually look like in a country where the epidemiological service and its communication with those in power works well, as it does in Singapore, Taiwan or South Korea. In Italy, we have unfortunately noticed the consequence of a free spread of the epidemic for too long. Such a development was not predictable. The biggest surprise of this pandemic to date to the community of epidemiologists is, undoubtedly, the lack of response by the authorities of many EU countries to the apparent spread of the pandemic at an exponential rate for weeks, leading to a very large numbers of infected people in a very short time. It is even more surprising that, although the Italian example exposed this problem quite clearly, a similar scenario is now happening in several other European countries.

How and why could this happen in Italy and then in other countries in the European Union (EU)? I will try to offer at least some hypotheses. First, EU countries have been living in prosperity for decades, focused mainly on their economies. Aside from the economic questions, they have not had any challenges that they’ve had to answer to swiftly and decisively, that would measure up to this one. Back in the 1960s, vaccines were introduced against most major infectious diseases, especially childhood ones. Malaria is no longer present in Europe and tuberculosis has been treated similarly for decades. The challenge of HIV/AIDS in the 1980s is now being successfully controlled with antiretroviral drugs. Liver inflammation is treated mainly by clinicians. The impact of influenza is controlled through vaccination, while rare zoonoses are resolved with immunoprophylaxis. Even sexually transmitted infectious diseases (STDs) are no longer as significant since the vaccine for Human Papillomavirus (HPV) was licensed.

The last real epidemic that concerned Europe was the Hong Kong flu, which occurred back in 1968 and 1969. The broad field of biomedicine offers such a wide range of exciting career paths to all those students who study it these days, but the epidemiology of infectious diseases is not really one of them – or, at least it hasn’t been in Europe for a very long time. It has probably begun to seem like an archaic medical profession to the majority of students and young medical doctors. It seemed to belong to the past for the European continent, which made it one of the least attractive things to specialize in.

Even the rare epidemiologists who specialized in infectious diseases have begun retraining for chronic non-communicable diseases, due to the aging of Europe’s population, which is particularly the case in Italy and Spain. It seems that at least some EU countries may have fallen victims to their own, decades-long success in the fight against infectious diseases. They faced this unexpected pandemic with few experts that could have had any experience in these events. Asian countries, as well as Canada, have had enough recent experience with SARS and MERS, but some European countries seem to have forgotten how to fight infectious diseases. If it were not for the legacy of the great Croatian epidemiologist, social medicine expert and global public health pioneer Dr Andrija Štampar, and the relatively recent war in Croatia, it is difficult to say whether or not Croatia would be as ready as it has proven itself to be.

Another factor that would understandably undermine the Italian response was that no one before Italy, in fact, could have observed how fast COVID-19 was spreading among the population. The greatest danger of COVID-19 is its accelerated, exponential spread when it breaks through the first line of defence. But, before Italy, only the Chinese in Wuhan and the Iranians had experienced the free spread of the infection among their population. After five days of monitoring the number of infected, the Chinese had to quarantine the entire Wuhan, and then further 15 cities a day later, in order to contain the virus. They did not know how many infected people were there outside of Wuhan’s hospitals. For Iran, however, only a few knew exactly what was happening, as that country is significantly isolated internationally due to political reasons. The Koreans, however, had a limited local epidemic but not an uncontrolled free spread — they caught the virus using their first line of defence.

That is how the Italians ended up becoming the first country in the highly developed world to monitor their epidemic spreading uncontrollably among the population. The only estimate of the rate of spread of the virus to date has been in the scientific work of Li et al. from the 29^th^ of January [23]. However, it was difficult to subsequently determine R0 parameter on the first 425 patients in Wuhan. The estimate of the R0 for COVID-19 in their article was 2.2, but with a very wide confidence interval – from 1.4 to 3.9. It is a bit of tough luck for the Italians, again, that they calculated the lower bound of the confidence interval to be 1.4 exactly. This figure is well known to all epidemiologists, it’s the rate of the spread of seasonal flu in the community. It should come as no surprise that many epidemiologists would guess that, with more data, R0 for COVID-19 would start converging more towards 1.4. Unfortunately, the more recent data suggests that R0 is more likely to lean towards 3.9, implying an incredibly fast spread. Thus, the greatest danger of COVID-19 remained unrecognized in Italy until the 8^th^ of March quarantine measures. At least 100 times fewer people would be dying in Italy these days had they declared a quarantine for Lombardy two weeks earlier than they did.

Just a few days ago, an extremely useful piece of scholarly work, authored by Onder et al., became available [24]. Their contribution finally provided answers to the three great unknowns about COVID-19. Many hypotheses have been presented in the media to explain the developments in Italy since the very outbreak of the epidemic, but thanks to just one simple table, we can now dispel most of them.

The first is the question that has plagued us for a long time – how dangerous is COVID-19 for younger age groups? It is clear that the media will tend to single out individual cases of death in younger people, as they are of most public interest. However, it is interesting that until recently, we didn’t really have decent data on this in the public domain. The first reason was that the Chinese Centre for Disease Control reported all deaths in the Chinese epidemic using age groups structure that contained a very large age group of “30-79 years”. It only separated children up to 10 years, then adolescents up to 20 years, then 20-29 year-olds, followed by this broad age group (30-79 years) and those who were 80 years or older. That is why the work of Onder and colleagues is commendable, as they subdivided this large group from China into 10-year age groups. This finally allowed a comparison between the first 1023 deaths in Wuhan (up to the 11^th^ of February) with the first 1625 deaths in Italy (up to the 17^th^ of March). The comparison is shown in **Table 2**.It gives us some very important insights.

**Table 2:** Age distribution of deaths from COVID-19 in Italy and China*

	Italy (up to March 17), an analysis of 1625 deaths		China (up to February 11), an analysis of 1023 deaths	
	**Number (%)**	**Case-fatality rate**	**Number (%)**	**Case-fatality rate**
0-9	0	0	0	0
10-19	0	0	1 (0.1%)	0.2%
20-29	0	0	7 (0.7%)	0.2%
30-39	4 (0.3%)	0.3%	18 (1.8%)	0.2%
40-49	10 (0.6%)	0.4%	38 (3.7%)	0.4%
50-59	43 (2.7%)	1.0%	130 (12.7%)	1.3%
60-69	139 (8.6%)	3.5%	309 (30.2%)	3.6%
70-79	578 (35.6%)	12.8%	312 (30.5%)	8.0%
80+	850 (52.3%)	20.2%	208 (20.3%)	14.8%

*Adapted from Onder et al. [24].

First, in Italy, more than half of the deaths initially were among people who were older than 80 years of age, and a total of 88% of the deaths occurred among the persons over 70 years of age. So, contrary to the impression that individual media reports can easily make, COVID-19 is a very dangerous disease mainly for old people. Moreover, a recent study by A. and G. Remuzzi [25] showed that, among 827 deaths in Italy, the vast majority of those people were already severely ill with underlying diseases, such as cardiovascular disease, diabetes, and malignancies. This is what epidemiologists expected because a more severe flu would have had a similar effect if there was no vaccine available. However, I doubt that the general public has the proper insight into this issue from the prevalent media reports.

Second, it was suggested in the media across Europe that the virus in Italy may have mutated and become much more dangerous. However, **Table 2** shows that death rates by the age of 70 are practically the same in China and Italy. Then, although the case fatality rate appears to be about 50% greater in Italy than in China for the age group 70-79, this does not suggest that the virus may have mutated. It is known that in Wuhan, many of the affected with a severe clinical presentation of COVID-19 could rely on the two newly built hospitals and respiratory aids that the military had brought in from other parts of China [26]. They also had medical teams coming in from other provinces [26]. In Italy, however, there were not enough respirators for this age group, and there weren’t enough doctors either, as many of them themselves became infected [16,20]. For those two reasons I would, in fact, expect even a larger difference between Italy and China than the one we are seeing, so I would not attribute this observed difference to the impact of the virus itself. And finally, the reported difference in case-fatality rates for the oldest age group should also not be attributed to the virus. It is more likely a consequence of the fact that Italians of Lombardy live, on average, longer than the Chinese of Wuhan [27]. Therefore, there are significantly more people in the oldest age group in Italy, ranging to much higher ages, so the two oldest groups are not really comparable. The average age of the Italians in the age group “80 years or older” is significantly greater than the average age of the oldest Chinese age group. Therefore, the table shows practically equal death rates across all age groups, on sufficiently large samples, meaning that the virus did not mutate in Italy from the virus we see from Wuhan, at least not until the 17^th^ of March, 2020.

Third, and perhaps most importantly — this chart has now made it quite clear that COVID-19 does not, in fact, kill people under the age of 50 unless they have some sort of underlying disease, or some unknown “Achilles heel” in their immune system that makes them particularly susceptible to the virus. There are such cases with every infectious disease. They are also present during the flu epidemics, but they are extremely rare. This suddenly gives us another possible strategy for quarantine exit, where children and those under 50 years of age could first emerge if they do not have any underlying illnesses. Here, after this table, it already seems like we are beginning to have an increasing number of options to get out of quarantines and learn to live with this virus until the vaccine becomes available. However, at least a few more studies need to be carried out to confirm that this age group can be substantially protected, to provide reassurance that the virus is not becoming more dangerous for those younger than 50 years, too.

There is another strangeness to the situation in Italy that will not be intuitive to the general public. The actual number of deaths attributable to COVID-19 in Italy will not be possible to estimate for several months after the epidemic finally ends. Namely, at present, due to the sole focus on the epidemic, most of the cases of death of very old people who have been diagnosed using a throat swab have been attributed to COVID-19. However, once the epidemic is over, it will be necessary to compare the deaths in individual areas of Italy with the average for the same months in the previous few years. It could be shown that a part of the already ill would have died in the same month, or a year, even without being infected with the new coronavirus. It is possible that among some of them COVID-19 accelerated this inevitability by a few weeks or months. Some of the deaths observed during the epidemic in Italy may need to be reclassified later and attributed to underlying diseases in accordance with expected levels, and those above expected levels will then be attributed to COVID-19.

This article provides an explanation from the epidemiologist’s point of view for most of the events reported so far in Italy, and then followed in Spain and other European countries. A combination of an early relaxation over this epidemic, systematic lack of expertise in the field of epidemic infectious diseases in Europe, likely inexperience in containing epidemic spread in this generation of health workers, relaxed immigration regulations for workers in certain industries, Champions League football event and a series of further misfortunes and omissions have all been implied so far in the media as contributors to the late withdrawal of Lombardy into quarantine. This allowed, through exponential growth, for a large number of people to be infected in a very short time – far too large for health systems of Italy and other EU countries to cope with. Severe forms of illness led to many deaths due to respiratory failure. In 88% of cases, people over 70 years of age died, who had underlying illnesses in the large majority of cases. This viewpoint is based on the first 1625 deaths in Italy, and by the time of this writing, there are now more than 10 000 dead. Given the size of the population, this would correspond to 670 deceased in Croatia. If Croatia manages not to follow the numbers of deceased seen in Italy, this will mostly be attributable to social exclusion measures being introduced early enough for its health system to come with severe cases.

These days, the people of Italy, Spain and other European countries are suffering large losses. A significant underlying cause may be the problem that the human brain simply cannot intuitively grasp the power of exponential growth, nor that two weeks of delay could make the difference between 100 and 10 000 deaths. Any physics enthusiasts will know the quote attributed to the great Albert Einstein, who is often cited saying that compound interest, which leads to exponential growth, is “…the most powerful force in the Universe”, or “…the eighth wonder of the world. He who understands it, earns it; he who doesn’t, pays it.” [28].
